# The overlooked relationship between motivational abilities and posttraumatic stress: a review

**DOI:** 10.3402/ejpt.v3i0.18560

**Published:** 2012-10-31

**Authors:** Keti Simmen-Janevska, Veronika Brandstätter, Andreas Maercker

**Affiliations:** Department of Psychology, University of Zürich, Zürich, Switzerland

**Keywords:** Motivation, PTSD, trauma, self-efficacy, locus of control, self-esteem, impulsivity

## Abstract

How does traumatic stress change the ability to motivate oneself to achieve certain goals? How do motivational abilities influence the development and course of trauma sequelae? Few studies have focused on motivational constructs within posttraumatic stress research. From a trauma research perspective, it can be hypothesized that traumatic stress may contribute to motivational dysfunction. The main goal of the present article is to fill this gap in research by reviewing and discussing the existing trauma literature in terms of motivation-related concepts, such as self-efficacy, locus of control, self-esteem, and self-control/impulsivity. Fifty-four studies were reviewed, 10 of which were longitudinal studies. Approximately 20% of the reviews assessed whether motivational concepts predict posttraumatic stress, whereas only 8% examined the reverse relationship. With the exception of a few studies, motivational constructs seem to predict posttraumatic stress over the life span. The strongest relationships were reported for self-efficacy, followed by locus of control and self-esteem and, lastly, impulsivity/self-control. Overall, the findings of this review indicate that there is a lack of research investigating motivational factors as outcome variables following traumatic experiences. Furthermore, the need for longitudinal studies and studies with older adults is noted.

Experiencing traumatic events, such as sexual abuse, natural disasters, or physical violence, often leads to serious changes in the psychological makeup of a person, particularly if a posttraumatic stress disorder (PTSD) develops. The traumatic sequelae include changes in motivation, cognition, and emotion.

Motivation is an umbrella term for a wide array of cognitive and affective processes that are involved in goal-directed behavior. Goals, which are defined as “internal representations of desired states, where states are broadly construed as outcomes, events, or processes” (Austin & Vancouver, 1996, p. 338), are at the center of motivational analysis (Bargh, Gollwitzer, & Oettingen, 2010). The pursuit of personally meaningful goals plays a pivotal role in individuals’ well-being and their life adjustments (Brunstein, Schultheiss, & Maier, 1999). There is abundant empirical evidence that progress in the achievement of personal goals relates to personal well-being (Brunstein, Schultheiss, & Grässmann, 1998). From this point of view, it seems of utmost importance to analyze whether traumatic stress affects factors known to be relevant to successful goal pursuit, as the inability to successfully strive for personal goals might aggravate the psychological condition of traumatized individuals.

Current motivational theorizing on goal striving distinguishes two related although conceptually distinct aspects, which are, on the one hand, selecting a goal from a variety of action alternatives (goal setting) and, on the other hand, pursuing the chosen goal vigorously (goal implementation) (Bargh et al., 2010). Goal setting is dependent on desirability and feasibility considerations, as illustrated, for example, in the influential expectancy-value-model of task choice (Atkinson, 1957) and in the theory of planned behavior (Ajzen & Fishbein, 1980). There are diverse constructs that can be subsumed under the concept of expectancy. More specifically, self-efficacy (Bandura, 1997), control beliefs (locus of control: Rotter, 1966), and self-esteem (Park, Crocker, & Kiefer, 2007) need to be high for strong goal commitments to emerge. Goal implementation, however, hinges on self-regulatory processes that are necessary to cope effectively with typical problems, such as initiating goal-directed actions (forming implementation intentions: Gollwitzer & Sheeran, 2006), persisting in the face of competing action tendencies (action control: Kuhl, 1984), and suppressing impulsive reactions (self-control: Hagger, Wood, Stiff, & Chatzisarantis, 2010).

Presently, goal-directed behavior has been studied in various psychological domains; however, most domains are outside of abnormal or clinical psychology, with the exception of studies regarding obsessive compulsive disorder (Woody & Szechtman, 2011) or treatment studies (Chard, Schumm, Owens, & Cottingham, 2010). In general, studies have reported a beneficial relationship between motivational components and psychological well-being (Forstmeier & Maercker, 2008) and favorable health behaviors (McAuley et al., 2007).

In the context of trauma, individuals respond to a traumatic event with intense fear, helplessness, or horror, and they perceive the stressor as uncontrollable or unpredictable (Foa, Zinbarg, & Rothbaum, 1992). Undergoing such uncontrollable adversities can lead to learned helplessness (Seligman, 1975) and, consequently, negatively influence outcome expectations. From a psychotraumatological perspective, for example, we can assume that a woman who has become a victim of chronic sexual abuse will show diminished initiative to actively respond to life requirements in comparison to a non-traumatized person of the same age. Moreover, she may be unable to learn from successful experiences, engage in less or restricted future planning, and develop affective deficits, such as anxiety or depression. A reformulated module of the original helplessness hypothesis has been supplemented with an attributional framework (Abramson, Seligman, & Teasdale, 1978). In particular, stable, global, and internal attributions determine the occurrence of helplessness after an adverse event. To illustrate, a war veteran who has witnessed mass killing may consider himself a loser (internal attribution) who will fail to fulfill any future intentions (global attribution) due to a lack of personal abilities (stable attribution). This personal helplessness will result in low self-esteem.

Researchers have argued that helplessness has the greatest impact on motivational deficits (Maier & Seligman, 1976). As helpless individuals show diminished expectancies for future success, their behavior may result in delayed proactive intentions (Abramson et al., 1978), which, in turn, may have a negative impact on goal setting and goal implementation. A closer look at the PTSD syndrome reveals that the symptoms in the avoidance/numbing cluster are motivational in nature, particularly “marked diminished interest in significant activities” (B4) and a “sense of a foreshortened future” (e.g., “does not expect to have a career, marriage”) (B7). All of these symptoms help the traumatized individual to escape from his or her painful past (Foa et al., 1992).

Given that helplessness is an important factor in the context of trauma, we would assume that a loss of control can influence motivational deficits by diminishing the subjective expectancies to steer future personal undertakings. Although the etiology research is particularly interested in whether motivation influences PTSD, the life-span research focuses more on whether PTSD or traumatic stress has an impact on motivation and goal striving. Therefore, the aim of the present review is to summarize the existing findings in the area of trauma/PTSD and motivation. For this reason, we chose a life-span approach and compiled relevant results from childhood to older adulthood.

## Outline of the included motivation-related constructs

Uncontrollable traumatic events may result in learned helplessness and, consequently, promote motivational deficits. Because subjective expectancies represent core competencies with regard to goal-directed behavior, it is possible that individuals affected by traumatic stress often lack motivational abilities. Accordingly, the motivational phenomena included in this review focus on the constructs that are related to goal setting and goal implementation and are frequently discussed in the trauma literature.

In trauma research, a number of studies have been conducted examining the motivational aspects of self-evaluation, such as self-efficacy, locus of control, and self-esteem (Judge, Erez, Bono, & Thoresen, 2002). We acknowledge that self-esteem can be considered a psychological construct that does not necessarily lead directly to goal attainment (Bandura, 1997). Nevertheless, self-esteem has frequently been linked to promoting motivation and effective behavioral outcomes (Pyszczynski, Greenberg, Solomon, Arndt, & Schimel, 2004). Self-control is important for goal implementation (Hagger et al., 2010) and has played a significant role in trauma research as well. We excluded motivation-related concepts that were not considered core concepts in motivational psychology, but that have been debated in traumatic stress research, including changed life priorities (Calhoun & Tedeschi, 2006), self-enhancement (Bonanno, 2004), existential goal seeking (Reker & Woo, 2011), hardiness (Kobasa, 1979), or optimism (Scheier, Carver, & Bridges, 1994).

Although measures of the self-efficacy, locus of control, and self-esteem constructs share common variance, and it is recommended to integrate the literature on these constructs (Judge et al., 2002), we decided to use the traditional separation structure. In choosing this approach, we highlight the variance that is unique to each construct. In addition, the separation represents the actual state of the art in which these constructs are treated in trauma research.

### Self-efficacy

Bandura (1997) defined the construct of self-efficacy as one's belief in one's ability to succeed in specific situations. Such beliefs influence motivational regulation, so that individuals model their own goals by investing an effort toward the expected outcome. Self-efficacy can be task- and situation-specific. A number of researchers understand self-efficacy as the self-evaluation of personal coping capabilities to manage environmental requirements following a traumatic event and refer to this as coping self-efficacy (Benight & Bandura, 2004). Other researchers conceptualize self-efficacy as a generalized conviction in being able to fulfill demanding requirements in a wide range of domains (Schwarzer & Jerusalem, 1995).

### Locus of control

This construct refers to the extent to which individuals believe that they can control events that affect them (Rotter, 1966). Two forms of locus of control are traditionally distinguished: internal and external. An internal locus of control implies that one's own behavior is seen as the main controlling factor for events in life. In contrast, an external locus of control implies that outcomes in one's life are due to chance, luck, fate, or are influenced by other powerful individuals.

### Self-esteem

Self-esteem is the personal appraisal of one's own self-worth (Fleming & Watts, 1980), which has a multidimensional nature and can vary in different life domains (Bandura, 1997). Interpersonal or man-made traumatic events, such as acts of extreme humiliation, primarily serve to intentionally reduce self-esteem (Baumeister, Smart, & Boden, 1996). However, it is conceivable that self-esteem declines in the context of accidental traumas, such as natural disasters, and may be influenced by survivor guilt, which many trauma victims experience (Hull, Alexander, & Klein, 2002).

### Impulsivity/self-control

Impulsivity has been established as the counterpoint to self-control, with many researchers treating these constructs as two sides of the same coin (Hofmann, Friese, & Strack, 2009). Impulsivity is defined as the tendency to execute actions imprudently and rapidly, without considering their possible negative consequences (Moeller, Barratt, Dougherty, Schmitz, & Swann, 2001). Studies have shown that having high levels of self-control is related to lowered likelihood of psychopathology (Tangney, Baumeister, & Boone, 2004).

### Aim of the review

To our knowledge, no review article is available that addresses the full range of specified motivation-related concepts in the trauma literature. The findings from Luszczynska, Benight and Cieslak's (2009) review article show strong cross-sectional and longitudinal associations between self-efficacy and general distress (e.g., anxiety and depression) and PTSD in adults and adolescents with exposure to a collective trauma. Given that those authors focused exclusively on the association between self-efficacy and psychological/somatic outcomes after a collective trauma, the main goal of our review is to go one step further and present and summarize the empirical evidence concerning the role of self-efficacy, locus of control, self-esteem, and impulsivity in individuals who have experienced individual or collective traumatic events over their lifespan. Four studies (Benight et al., 1997; Benight & Harper, 2002; Saigh, Mroueh, Zimmerman, & Fairbank, 1995; Sumer, Karanci, Kazak Berument, & Gunes, 2005) that were reviewed by Luszczynska et al., (2009) will also be analyzed in the present article, as these articles met our selection criteria.

The present article aims to synthesize the empirical findings regarding the relationship between motivational variables and traumatic stress. Based on extant theory, the main assumption of the current review is that traumatic stress should decrease motivational capacities. First, we will investigate whether longitudinal studies have been conducted and, if so, whether these studies allow us to draw conclusions about the direction of the effects. We will then examine the function of the four constructs on which research has largely focused, primarily examining their roles as predictors and mediators, as well as outcomes. Finally, we will investigate gender-related effects.

## Method

For this review, studies published in peer-reviewed journals prior to August 2011 were selected through a computerized literature search using the PsychINFO, PubMed, and PILOTS databases. The included articles examined the direct or indirect relationship between motivational variables and traumatic distress/adversities across the life span. Initially, we performed a search using a combination of the terms “trauma*/advers*” and terms for every motivational aspect. More precisely, we combined the terms “trauma*/advers*” with “self-efficacy”, “locus of control”, “self-esteem”, and “impuls*”. To limit the number of search results, we only considered studies that contained these terms either in the title or in the abstract. This procedure generated over 700 studies. Next, we scanned the abstracts of the remaining studies and included only those that were quantitative and published in English language journals. Moreover, we made no restrictions in terms of age of the sample, type of trauma, and nature of the motivational variables (e.g., general self-efficacy or coping self-efficacy). In addition, studies that covered a wide range of physical and mental health outcomes were reviewed. Excluded were therapeutic intervention studies and studies that dealt with resilience or exclusively with medical concerns. Studies with traumatized subjects that included one motivation-related construct, but did not test the association between traumatic stress/PTSD and that specific construct, were eliminated from this analysis. The final search yielded 54 articles to be critically reviewed.

## Results

### Overview of relevant studies

In total, 54 studies were identified as relevant (Table 1). Approximately one-fifth of the studies had a longitudinal design. Most studies dealt with the construct self-esteem, within which all but one study had a cross-sectional design.

**Table 1:** Number of studies arranged by research design

Construct	Longitudinal	Cross-sectional	Subtotals
Self-efficacy	8	7	15
Locus of control	1	14	15
Self-esteem	1	19	20
Impulsivity	0	9	9

*Note*: Five studies have been counted double.

The majority of the studies (approx. 54%) included samples of young adults aged 22 to 40 years, followed by samples of children and adolescents aged between 0 and 21, which accounted for approximately one-third of the studies. Nine studies considered samples with middle-aged individuals aged 41 to 60 and only two studies examined data from samples with adults aged 60 and older.

To obtain a more comprehensive picture of the trauma type, Table 2 lists the data provided in the literature reviewed. For this purpose, we have chosen the categorization proposed by Luz et al. (2011). As a result, individual traumas clearly predominate, specifically child abuse, followed by war-related traumas and natural disasters.

**Table 2:** Categorization of traumatic events

	Self-efficacy	Self-esteem	Locus of control	Impulsivity	Total
Collective trauma	8	5	10	0	23
War-related trauma	2	2	5	–	9
Natural disaster	4	2	2	–	8
Chronic complex exposure in high-risk professions	2	–	3	–	5
Terrorism	–	1	–	–	1
Individual trauma	7	15	5	9	36
Child abuse	2	8	3	5	18
Medical causes	2	3	–	–	5
Mixed trauma population	–	2	–	2	4
Child abuse/non-A1 event	–	1	1	1	3
Injury	–	–	1	1	2
Insufficient information	1	1	–	–	2
Violent crime	1	–	–	–	1
Motor vehicle accident	1	–	–	–	1

*Note*: Five studies examined more than one concept and were counted double (war-related, natural disaster, chronic complex exposure, child abuse/non-A1 event, insufficient information).

### Findings from longitudinal studies

The findings from 10 longitudinal studies are presented in Table 3.

**Table 3:** Longitudinal findings

Authors	Construct	Sample	Design	Main Finding
Benight, Cieslak, Molton, & Johnson, 2008	CSE (predictor, mediator)	Motor vehicle accident (*N*=70) Adulthood Mixed gender (*F*=63%)	1 group Assessment at approx. 7, 30, and 90 days post-event	*r*= − 0.57*** between CSE (T1) and PTSD (T3) *R* ^2^=0.19 change CSE (T1 to T2), PTSD (T1) and involvement in litigation predict PTSD (T3) CSE (T2) mediates relation between PTSD (T1) and PTSD (T3) η^2^=0.36 CSE increases from T1 to T2 No gender differences regarding CSE
Johansen, Wahl, Eilertsen, & Weisaeth, 2007	SE (predictor)	Non-domestic violence (*N*=70) Adulthood Mixed gender (*F*=17%)	1 group Assessment at few days – 16 weeks, 3, and 12 months post-event	*r*= − 0.44*** between SE (T1) and PTSD (T2) and *r*= − 0.25* between SE (T1) and PTSD (T3) No change in SE from T1 to T3
Heinrichs et al., 2005	SE (predictor)	Firefighters (*N*=43) Early adulthood Male	1 group Assessment at baseline, 6, 9, 12 and 24 months post-training	*R* ^2^=0.42 SE (T1) and hostility (T1) predict PTSD (T5) No change in SE from T1 to T5
Wolfe, Wekerle, Scott, Straatman, & Grasley, 2004	SE (predictor)	Dating violence (*N*=1317) Adolescence Mixed gender (*F*=55%)	1 group Assessment at baseline and 12 months post-event	SE (T1) does not predict dating violence (T2) in boys and girls SE stable from T1 to T2 for both genders
Benight & Harper, 2002	CSE (predictor, mediator)	Natural disaster (*N*=46) Middle adulthood Mixed gender (*F*=41%)	1 group Assessment at 3– 8 weeks and 12 months post-event	*r*= − 0.65** between CSE (T1) and PTSD (T2) *R* ^2^=0.07 CSE (T1) and PTSD (T1) predict PTSD (T2) CSE (T1) mediates relation between acute stress (T1) and PTSD (T2) and between acute stress (T1) and general psychological distress (T2) Lower CSE in females
Koopman et al., 2002	SE (predictor)	Breast cancer (*N*=117) Adulthood Female	1 group Assessment at baseline and 6 months post-diagnosis	SE (T1) does not predict PTSD (T2)
Solomon, Benbenishty, & Mikulincer, 1991	SE (outcome)	Frontline soldiers (*N*=65) Early adulthood Male	1 group Assessment at 12, 24 and 36 months post-event	*R* ^2^=0.60 psychic numbing in combat (T1), psychopathology (T2) and pre-military adjustment (T1) predict SE (T2) No change in SE from T1 to T3
Murphy, 1988	SE (predictor)	Natural disaster (*N*=101) Early adulthood Mixed gender (*F*=70%)	1 group Assessment at baseline and 24 months post-event	*R* ^2^=0.19 SE (T1) predicts general distress (T2) *R* ^2^=0.17 SE (T1) predicts depression (T2) *R* ^2^=0.12 SE (T1) predicts somatization (T2)
Lynch & Cicchetti, 1998	Self-esteem (outcome)	Childhood trauma (*N*=322) Childhood Mixed gender (*F*=37.3%)	2 groups (Maltreated vs. controls) Assessment at baseline and 12 months post-event	*r*= − 0.13* between childhood trauma (T1) and self-esteem (T2)
Solomon, Mikulincer, & Avitzur, 1988	LOC (predictor)	Frontline soldiers (*N =* 262) Early adulthood Male	1 group Assessment at 24 and 36 months post-event	Partial *r*= − 0.19** between LOC (T1) and PTSD (T2), controlled for PTSD (T1) Change in LOC from T1 to T2, LOC becomes more internal

*Note*: CSE = coping self-efficacy; SE = self-efficacy; LOC = locus of control; gender, F = female; childhood/adolescence = 0–21 years; early adulthood = 22–40 years; middle adulthood = 41–60 years; adulthood = from early to old adulthood (>60 years);

* *p*<.05

** *p*<.01

*** *p*<.001

Conclusions from eight longitudinal studies on self-efficacy are as follows. First, there is evidence for a moderate to strong negative longitudinal association between self-efficacy and PTSD (Benight et al., 2008; Benight & Harper, 2002; Johansen et al., 2007), meaning that individuals with high levels of self-efficacy experience less traumatic symptoms and, conversely, individuals with low self-efficacy report more traumatic symptoms. Second, the primary focus of the reviewed studies was the predictive role of self-efficacy on PTSD or overall distress after trauma exposure, which was confirmed in four samples (Benight & Harper, 2002; Heinrichs et al., 2005; Johansen et al., 2007; Murphy, 1988). That is, having a high or low degree of self-efficacy prior to the trauma seems to be related to lower or higher PTSD symptoms over time. Third, there is an indication that self-efficacy mediates the effect of acute stress/baseline PTSD on subsequent PTSD (Benight et al., 2008; Benight & Harper, 2002). Expressed differently, self-efficacy may determine whether acute stress immediately after trauma influences the progression of PTSD over time. Fourth, one study revealed the reverse effect of traumatic stress predicting self-efficacy (Solomon et al., 1991). Finally, self-efficacy appears to remain stable over time (Heinrichs et al., 2005; Johansen et al., 2007; Solomon et al., 1991). However, this finding from three studies should be viewed with caution, as the factors contributing to self-efficacy may be different across studies.

With regard to locus of control, one study concluded that an internal locus of control predicts a decrease in PTSD intensity (Solomon et al., 1988), which suggests that individuals who are convinced that they can control their life events will suffer less from PTSD symptoms.

The only longitudinal study on self-esteem reported a weak negative correlation between self-esteem and traumatic stress in children (Lynch & Cicchetti, 1998). Specifically, subjects with favorable appraisals of their self-worth seemed to be less affected by PTSD.

### Findings from cross-sectional studies

The results of cross-sectional studies are organized in Tables 4, 5, 6, and 7. We use the same structure that was applied by the authors in the original articles, namely, the organization by predictor, mediator, and outcome. Given the cross-sectional nature of the studies, we emphasize that this approach is used to simplify the presentation of the studies’ results and is not intended to convey an assumption of causal relationships.

**Table 4:** Self-efficacy: cross-sectional findings

Authors	Sample	Design	Main Finding
Bender, Ferguson, Thompson, Komlo, & Pollio, 2010	Homelessness (*N*=146)^1^ Early adulthood Mixed gender (*F*=37%)	1 group	Lower SE in individuals with PTSD vs. no PTSD
Kohno et al., 2010	Gastrointestinal cancer (*N*=47) Adulthood Mixed gender (*F*=32%)	1 group	*r*=−.36* between SE and posttraumatic stress symptoms
Cieslak, Benight, & Caden Lehman, 2008 (Study 1)	Childhood trauma (*N*=66) Early and middle adulthood Female	1 group	*r*=−.57*** between CSE and posttraumatic distress CSE mediates relation between negative cognitions about self/world and posttraumatic distress
Sumer, Karanci, Kazak Berument, & Gunes, 2005	Natural disaster (*N*=350) Adulthood Mixed gender (*F*=52%)	1 group	*R*=−.24*** between SE and intrusion and *r*=.03 between SE and avoidance SE does not mediate relation between trauma exposure and psychological distress Lower SE in females
Wolfe et al., 2004	Dating violence (*N*=1317) Adolescence Mixed gender (*F*=55%)	1 group Assessment at baseline and 12 months post-event	No significant *r* (not reported) between SE (T1) and child maltreatment (T1) in boys *r*=−.17*** between SE (T1) and child maltreatment (T1) in girls
Koopman et al., 2002	Breast cancer (*N*=117) Adulthood Female	1 group Assessment at baseline and 6 months post-diagnosis	*r*=−.27** between SE (T1) and impact of illness (T1) *R* ^2^=.34 SE (T1) and postsurgical treatment (T1) predict PTSD (T1)
Regehr, Hill, & Glancy, 2000	Firefighters (*N*=164) Adulthood Male	2 groups (Professionals vs. volunteers)	*r*=−.18* between SE and posttraumatic stress symptoms
Benight et al., 1997	Natural disaster (*N*=79) Early and middle adulthood Male	2 groups (HIV-Infected vs. controls)	*r*=−.70** between CSE and PTSD in HIV-Infected *r*=−.55** between CSE and PTSD in Controls *R* ^2^=.51 SE predicts PTSD in HIV-Infected *R* ^2^=.27 SE predicts PTSD in Controls
Saigh, Mroueh, Zimmerman, & Fairbank, 1995	War (*N*=30) Childhood Mixed gender (*F*=37%)	3 groups (PTSD+, traumatized PTSD-, controls)	Lower SE in individuals with PTSD vs. no PTSD

*Note*: ^1^=Trauma type not specified; SE = self-efficacy; CSE = coping self-efficacy; gender F = female; childhood/adolescence = 0–21 years; early adulthood = 22–40 years; middle adulthood = 41–60 years; adulthood = from early to old adulthood (>60 years);

* *p*<.05

** *p*<.01

*** *p*<.001

**Table 5:** Locus of control: cross-sectional findings

Authors	Sample	Design	Main Finding
Mellon, Papanikolau, & Prodromitis, 2009	Natural disaster (*N*=800) Adulthood Mixed gender (*F*=47.9%)	2 groups (Affected areas vs. non-affected areas)	*r*=.15*** between external LOC and traumatic stress in Affected Areas *r*=.05 between external LOC and traumatic stress in Non-Affected Areas
Al-Turkait & Ohaeri, 2008	Military men (*N*=200) Adulthood Male	4 groups (Retired vs. active-in-army vs. involved-in-combat vs. prisoners of war)	*r*=.20* between external LOC and traumatic stress No difference in LOC between groups
Klensmeden Fosse & Holen, 2007	Childhood trauma (*N*=160) Early adulthood Mixed gender (*F*=67%)	1 group	*R* ^2^=.09 emotional neglect and bullying by peers predict external LOC
Chung, Preveza, Papandreou, & Prevezas, 2006	Spinal cord injury (*N*=116) Middle adulthood Mixed gender (*F*=33.6%)	2 groups (Spinal cord injury vs. controls)	Adjusted *R* ^2^=.13 internal LOC predicts re-experiencing Adjusted *R* ^2^=.20 internal LOC predicts avoidance
Kuterovac-Jagodic, 2003	War (*N*=252) Childhood Mixed gender (*F*=49%)	1 group	*r*=.39*** between external LOC and traumatic stress *R* ^*2*^=.36 LOC, exposure to violence, expressive coping, social support and age predict PTSD Higher external LOC in boys
Maercker & Herrle, 2003	War (*N*=47) Middle to old adulthood Mixed gender (*F*=81%)	1 group	*r*=.46** between external LOC and intrusions, *r*=.51** between external LOC and avoidance, *r*=.54** between external LOC and hyperarousal *r*=.36 * between internal LOC and avoidance, *r*=.21 between internal LOC and intrusions, *r*=.14 between internal LOC and hyperarousal External LOC mediates relation between trauma exposure and PTSD Internal LOC moderates relation between trauma exposure and PTSD
Brown, Mulhern, & Joseph, 2002	Firefighters (*N*=248) Adulthood Male	1 group	*R*=.05 between LOC and traumatic stressors
Simoni & Ng, 2002	Childhood trauma (*N*=222) Adulthood Female	1 group	LOC does not mediate relation between childhood trauma and adult physical health
Brown, Mulhern, & Joseph, 2002	Natural disaster (*N*=130) Early adulthood Mixed gender (*F*=38.5%)	2 groups (Affected Areas vs. non-affected areas)	Higher external LOC in individuals with traumatic stress vs. no traumatic stress
Regehr et al., 2000	Firefighters (*N*=164) Adulthood Male	2 groups (Professionals vs. volunteers)	*r*=−.03 between internal LOC and traumatic stress
Kilpatrick & Williams, 1998	Domestic violence (*N*=35) ChildhoodMixed gender (*F*=48.6%)	2 groups (Exposed vs. controls)	LOC does not predict PTSD
Weiss, Marmar, Metzler, & Ronfeldt, 1995	Emergency services personnel (*N*=367) Early adulthood Mixed gender (*F*=10.6%)	2 groups (Exposed to natural disaster vs. controls)	*r*=.23** between external LOC and PTSD
Hall, Bolen, & Webster, 1994	Childhood trauma (*N*=315) Early adulthood Mixed gender (*F*=70%)	3 groups (Exposed to parental alcoholism vs. exposed to other trauma vs. controls)	No difference in LOC in individuals with and without traumatic stress
Solomon et al., 1988	Frontline soldiers (*N*=262) Early adulthood Male	1 group Assessment at 24 and 36 months post-event	*r*=−.38** between internal LOC (T1) and PTSD (T1)
Hyer, Boudewyns, O’Laery, & Harrison, 1987	War (*N*=75) Early adulthood Gender not reported	1 group	*R* ^2^=.75 external LOC and current stress level predict PTSD

*Note*: LOC = locus of conrol; gender, F = female; childhood/adolescence = 0–21 years; early adulthood = 22–40 years; middle adulthood = 41–60 years; adulthood = from early to old adulthood (>60 years);

* *p*<.05

** *p*<.01

*** *p*<.001

**Table 6:** Self-esteem: cross-sectional findings

Authors	Sample	Design	Main Finding
Grasso et al., 2011	Various potentially traumatic events (*N*=3119) Adolescence Mixed gender (*F*=45%)	1 group	*R* ^2^=.10 self-esteem and social support predict PTSD
Kuo, Goldin, Werner, Heimberg, & Gross, 2011	Childhood trauma (*N*=132) Early adulthood Mixed gender (*F*=66.7%)	2 groups (Social anxiety disorder vs. controls)	*r*=−.44*** between self-esteem and emotional neglect *r*=−.35*** between self-esteem and emotional abuse *r*=−.06 between self-esteem and physical neglect *r*=−.13 between self-esteem and physical abuse *r*=−.06 between self-esteem and sexual abuse
Bender et al., 2010	Homelessness (*N*=146)^1^ Early adulthood Mixed gender (*F*=37%)	1 group	No difference in self-esteem in individuals with and without trauma exposure
Hotun Sahin et al., 2010	Childhood trauma (*N*=750) Adulthood Female	1 group	*r*=−.64*** between self-esteem and childhood trauma
Vigil, Gaery, Granger, & Flinn, 2010	Natural disaster (*N*=115) Adolescence Mixed gender (*F*=61.7%)	2 groups (Exposed vs. controls)	*r*=−.23* between self-esteem and traumatic stress Lower self-esteem in males
Adewuya et al., 2009	HIV-stigma (*N*=190) Adulthood Mixed gender (*F*=54.7%)	1 group	Self-esteem, past traumatic events, social support and general psychopathology predict PTSD
Boscarino & Adams, 2009	Terrorist attack (*N*=2368) Adulthood Mixed gender (*F*=53.8%)	1 group	Gender, ethnicity, self-esteem, negative life events, trauma exposure, lifetime traumatic events, handedness and depression predict PTSD
Li et al., 2009	Various potentially traumatic events (*N*=1625) Childhood Mixed gender (*F*=49%)	3 groups (AIDS orphans vs. vulnerable children vs. controls)	*r*=.00 to –.08 between self-esteem and trauma exposure scales occurrence, density, duration of impact, initial impact, lasting impact No gender differences regarding self-esteem
Al-Turkait & Ohaeri, 2008	Military men (*N*=200) Adulthood Male	4 groups (Retired vs. active-in-army vs. involved-in-combat vs. prisoners of war)	*r*=−.39*** between self-esteem and traumatic stress Lower self-esteem in individuals with PTSD vs. no PTSD
David, Ceschi, Billieux, & van der Linden, 2008	Various potentially traumatic events (*N*=132) Early adulthood Mixed gender (*F*=88%)	1 group	*r*=−.13 between self-esteem and traumatic events Self-esteem does not mediate relation between traumatic events and depression
Klensmeden Fosse & Holen, 2007	Childhood trauma (*N*=160) Early adulthood Mixed gender (*F*=67%)	1 group	*R* ^2^=.19 mother overprotection, bullying by peers and childhood sexual abuse predict self-esteem
Finzi-Dottan & Karu, 2006	Childhood trauma (*N*=196) Early and middle adulthood Mixed gender (*F*=66%)	1 group	*r*=−.44*** between self-esteem and emotional abuse *R* ^2^=.30 emotional abuse, parental control and maternal care predict self-esteem Self-esteem mediates relation between childhood emotional abuse and adult psychopathology
Kashdan, Uswatte, & Julian, 2006	War (*N*=77) Adulthood Mixed gender (*F*=1%)	3 groups (Outpatients vs. residential patients vs. controls)	Trait negative affect and trait gratitude predict self-esteem Lower self-esteem in individuals with PTSD vs. no PTSD
Sumer et al., 2005	Natural disaster (*N*=350) Adulthood Mixed gender (*F*=52%)	1 group	*r*=−.37*** between self-esteem and intrusion *r*=−.01 between self-esteem and avoidance *R* ^2^=.26 self-esteem and optimism predict self-efficacy *R* ^2^=.29 self-esteem, optimism, material loss, perceived threat and gender predict intrusion Lower self-esteem in females
Langeveld, Grootenhuis, Voute, de Haan, & van den Bos, 2004	Childhood cancer (*N*=960) Early and middle adulthood Mixed gender (*F*=50.8%)	2 groups (History of cancer vs. controls)	*R* ^2^=.08 to .37 self-esteem, gender, unemployment and health problems predict quality of life dimensions No difference in self-esteem in individuals with and without trauma exposure Lower self-esteem in females
Stein, Burden Leslie, & Nyamathi, 2002	Childhood trauma (*N*=581) Early and middle adulthood Female	1 group	*r*=−.18*** between self-esteem and childhood trauma Self-esteem mediates relation between childhood trauma and adult depression/substance abuse
Feiring, Taska, & Lewis, 1998	Sexual abuse (*N*=142) Childhood/adolescence Mixed gender (*F*=76.1%)	2 groups (Children vs. adolescents)	*r*=−.22** between self-esteem and number of traumatic events *R* ^2^=.31 age, number of abusive events, attributional risk and shame predict self-esteem No gender differences regarding self-esteem
Lynch & Cicchetti, 1998	Childhood trauma (*N*=322) Childhood Mixed gender (*F*=37.3%)	2 groups (Maltreated vs. controls)	*R* ^2^=.20 age, victimization by violence and parental neglect predict self-esteem Lower self-esteem in maltreated vs. non-maltreated children No gender differences regarding self-esteem
Bunce, Larson, & Peterson, 1995	Various potentially traumatic events (*N*=58) Early adulthood Mixed gender (*F*=69%)	1 group	Lower self-esteem in individuals with traumatic stress vs. no traumatic stress
Fox & Gilbert, 1994	Physical abuse (*N*=253) Early adulthood Female	1 group	Significant *r* (not reported) between self-esteem and physical abuse

*Note*: ^1^ Trauma type not specified; gender, F = female; childhood/adolescence = 0–21 years; early adulthood = 22–40 years; middle adulthood = 41–60 years; adulthood = from early to old adulthood (>60 years);

* *p*<.05

** *p*<.01

*** *p*<.001

### Self-efficacy

Table 4 presents the cross-sectional results regarding the relationship between self-efficacy and traumatic stress. The significant weak to strong negative correlations between self-efficacy and posttraumatic stress in adults varied from *r*= − 0.18 in professional firefighters (Regehr et al., 2000) to *r*= − 0.70 in survivors of a natural disaster (Benight et al., 1997). The findings from a student sample indicated no relationship between maltreatment in childhood and self-efficacy for boys, and a significant small association between those variables for girls (Wolfe et al., 2004). One study of adolescents (Saigh et al., 1995) and one of adults (Bender et al., 2010) revealed that individuals with lower levels of self-efficacy tended to meet the criteria for PTSD more often than did individuals with higher levels of self-efficacy. In addition, two studies examined the mediating role of self-efficacy (Cieslak et al., 2008; Sumer et al., 2005). In the first study (Cieslak et al., 2008), coping self-efficacy functioned as a mediator between negative cognitions and posttraumatic distress. In the second study (Sumer et al., 2005), although coping self-efficacy was significantly negatively related to general psychological distress and intrusion, it did not mediate the association between trauma exposure and psychological distress in survivors of an earthquake.

### Locus of control

Table 5 depicts the studies that included locus of control in their analyses. The cross-sectional findings regarding locus of control are inconsistent. The vast majority of the studies that we reviewed report a small to medium positive relationship between an external locus of control and traumatic stress (Al-Turkait & Ohaeri, 2008; Kuterovac-Jagodic, 2003; Maercker & Herrle, 2003; Mellon et al., 2009; Weiss et al., 1995). That is, individuals who do not feel in control over the events in their lives tend to be affected by posttraumatic symptoms to a greater degree. With regard to an internal locus of control, the findings are contradictory. One study reported a medium negative correlation in soldiers (Solomon et al., 1988) while other studies have found that an internal locus of control was significantly related to the PTSD symptoms of avoidance and re-experiencing, but not to hyperarousal (Chung et al., 2006; Maercker & Herrle, 2003). In contrast, some studies did not find a significant correlation between locus of control and posttraumatic distress in firefighters (Brown et al., 2002; Regehr et al., 2000) or in children with exposure to domestic violence (Kilpatrick & Williams, 1998). Furthermore, the mediator and moderator roles of locus of control still remain unclear (Maercker & Herrle, 2003; Simoni & Ng, 2002). Only one study examined the role of locus of control as an outcome and found that childhood adversities, especially emotional neglect and bullying by peers, explained variance in the external locus of control (Klensmedan Fosse & Holen, 2007).

### Self-esteem

Self-esteem has frequently been studied in trauma research, both in samples with children and adults. The results are shown in Table 6.

Findings from cross-sectional studies appear to be consistent in that there is a small to medium negative relationship between self-esteem and posttraumatic stress symptoms (e.g., Al-Turkait & Ohaeri, 2008; Li et al., 2009), indicating that subjects with higher self-esteem experience traumatic stress to a lesser extent. In addition, several studies have reported significant negative associations between self-esteem and childhood adversities (e.g., Stein et al., 2002; Vigil et al., 2010), with the highest negative association found between childhood emotional abuse/emotional neglect and self-esteem (Finzi-Dottan & Karu, 2006; Kuo et al., 2011). Three studies examined a mediation model regarding self-esteem, with two studies finding that childhood adversities had an indirect effect on adult psychopathology, as mediated by self-esteem (Finzi-Dottan & Karu, 2006; Stein et al., 2002), and one study finding no mediation of self-esteem on the relationship between traumatic events and depression (David et al., 2008). The results regarding group differences are mixed, with half of the studies finding group differences in self-esteem between individuals with and without PTSD. From a methodological perspective, these results may be misleading. Many researchers have pointed out that group comparisons originating from dichotomization of a variable (e.g., low and high self-esteem) can lead to false interpretations due to a loss in variance and analytical power (Cohen, 1983; DeCoster, Iselin, & Gallucci, 2009). Considering this issue, it is not compellingly evident that individuals with high self-esteem really experience fewer traumatic symptoms.

### Impulsivity/self-control

The cross-sectional findings are illustrated in Table 7.

**Table 7:** Impulsivity: cross-sectional findings

Authors	Sample	Design	Main Finding
Narvaez et al., 2012	Childhood trauma (*N*=84) Early adulthood Mixed gender (*F*=8%)	1 group	Hedges effect size *g*=.81 between impulsivity and childhood trauma
Fishbein et al., 2009	Childhood trauma (*N*=553) Childhood Mixed gender (*F*=52%)	1 group	No significant *r* (not reported) between impulsivity and traumatic distress
Moehler et al., 2009	Childhood trauma (*N*=119) Adulthood (not specified) Female	2 groups (Abused vs. controls)	Significant *r* (not reported) between impulsivity and childhood trauma
Ariga et al., 2008	Various potentially traumatic events (*N*=64) Adolescence Female	1 group	No significant *r* (not reported) between impulsivity and traumatic distress
Ledgerwood & Petry, 2006	Childhood trauma (*N*=149) Middle adulthood Mixed gender (*F*=52%)	2 group (Low vs. high PTSD)	Significant *r* (not reported) between impulsivity and childhood trauma
Willebrand, Kildal, Andersson, & Ekselius, 2002	Burn accident (*N*=166) Middle adulthood Mixed gender (*F*=20.5%)	1 group	Higher impulsivity in individuals with traumatic stress vs. no traumatic stress
Brodsky et al., 2001	Childhood trauma (*N*=136) Early adulthood Mixed gender (*F*=64%)	2 groups (Abused vs. controls)	Impulsivity does not mediate relation between childhood trauma and adult suicidality Higher impulsivity in individuals with traumatic stress vs. no traumatic stress
Kotler, Iancu, Efroni, & Amir, 2001	Various potentially traumatic events (*N*=46) Middle adulthood Mixed gender (*F*=24%)	3 group (PTSD vs. anxiety disorder vs. controls)	*R* ^2^=.58 impulsivity and social support predict suicide risk
Zlotnick et al., 1997	Childhood trauma (*N*=85) Adulthood Mixed gender (*F*=42%)	1 group	Significant *r* (not reported) between impulsivity and childhood trauma

*Note*: Gender, F = female; childhood/adolescence = 0–21 years; early adulthood = 22–40 years; middle adulthood = 41–60 years; adulthood = from early to old adulthood (>60 years).

The findings primarily suggest a significant relationship between childhood trauma and impulsivity among adults (Brodsky et al., 2001; Moehler et al., 2009; Narvaez et al., 2012; Zlotnick et al., 1997), although this has not been found in child samples (Ariga et al., 2008; Fishbein et al., 2009). Unfortunately, most of the studies did not report a correlation coefficient, so it is impossible to make statements about the strength of the relationship. There is some evidence in that some studies show participants with a traumatic experience show higher impulsivity than controls (Brodsky et al., 2001; Willebrand et al., 2002). This may also be explained by variance differences in correlational versus group comparison designs. Furthermore, one study failed to confirm the mediating role of impulsivity on the relationship between abusive experiences in childhood and later suicidality in adults with major depression (Brodsky et al., 2001).

### Gender effects

With regard to gender effects, few studies have assessed differences between male and female participants. With regard to self-efficacy, two studies (Benight & Harper, 2002; Sumer et al., 2005) have found that women report lower self-efficacy than men, whereas one study did not find any gender differences (Benight et al., 2008). Gender differences were examined in only one study assessing locus of control, which found that boys externalize to a higher extent than girls (Kuterovac-Jagodic, 2003). Inconsistent results have emerged regarding the concept of self-esteem as well. In studies with children, the level of self-esteem seems to be equal among girls and boys (Feiring et al., 1998; Li et al., 2009; Lynch & Cicchetti, 1998). Studies of young adults, however, have found lower levels of self-esteem in women (Langeveld et al., 2004; Sumer et al., 2005), although one study of adolescents found that males reported lower self-esteem than females (Vigil et al., 2010). Finally, none of the studies on impulsivity tested gender effects.

## Discussion and future directions

Taken together, the majority of the longitudinal studies reviewed here suggest that self-efficacy is a robust predictor of the extent or severity of posttraumatic stress in adulthood. The strongest associations between traumatic stress and motivational constructs were found for self-efficacy, followed by self-esteem and locus of control with approximately equally strong relations, and, finally, relatively inconsistent associations with impulsivity. This may reflect a continuum of the most- versus least-involved motivational processes in traumatization. Self-efficacy would, therefore, be the most powerful motivational component that is involved in the development of traumatic symptoms.

Our review supports the findings of Luszczynska et al. (2009) regarding the relationship between self-efficacy and traumatic distress. Due to a lack of research, we did not find evidence that traumatic stress affects self-efficacy. From a theoretical perspective, self-efficacy beliefs are determined by mastery experience (e.g., success raises self-efficacy), vicarious experience (e.g., success is produced by models), verbal persuasions (e.g., verbal judgments by others), and physiological states (e.g., anxiety and stress) (Bandura, 1997). Consequently, we hypothesize that experiencing posttraumatic stress would damage self-efficacy. However, the empirical evidence for this assumption is still lacking. In the literature reviewed, only three longitudinal studies investigated the development of self-efficacy and suggested that self-efficacy remains stable after a traumatic exposure. However, two of the three studies examined professional samples after a collective trauma (i.e., firefighters and soldiers) and one study assessed victims with an individual trauma (i.e., non-domestic violence). One possible explanation for this phenomenon is that the type of trauma experienced influences the level to which self-efficacy changes. Therefore, we would assume that individual traumas, especially man-made, exert the highest impact on self-efficacy.

The small to moderate and, in some studies, even absent association between locus of control and posttraumatic stress is surprising. Our main assumption was that perceiving uncontrollability would be associated with more PTSD symptoms. One explanation for the unexpected findings may be linked to the debate on self-blame (Davis, Lehman, Silver, Wortman, & Ellard, 1996) or on counterfactual thinking (Leithy, Brown, & Robbins, 2006) in trauma victims. For example, a rape victim's belief that he or she could have avoided the event by having acted differently is similar to the perception generated by an internal locus of control, according to which one's own behavior determines the events in one's life. Thus, self-blame or counterfactual thinking may serve as a mediator or moderator in the relationship between locus of control and traumatic stress. For this reason, future studies should further investigate the role of controllability in the trauma context.

One reason for the weak association between self-esteem and posttraumatic stress might lie in the understanding of the construct of self-esteem. Crocker and Park (2004) discussed the costs and benefits of pursuing self-esteem and concluded that pursuing self-esteem is only beneficial if the advantages pre-dominate the efforts. In other words, it is essential to detect what goals individuals are striving for to show to themselves and others that they are valuable and worthy people. The trauma research has primarily focused on the level of self-esteem. Consequently, it may be that pursuing self-esteem, or pursuing goals to achieve self-esteem, is more important than just the level of self-esteem in traumatized individuals.

The inconsistent findings regarding impulsivity in trauma victims are, in a way, not surprising. Given the fact that literature on impulsivity in trauma victims has often considered samples who suffer from a comorbid disorder (e.g., substance abuse or borderline personality disorder), it is possible that the association between trauma and impulsivity is confounded by other factors related to a concurrent psychiatric disorder (Marshall-Berenz, Vujanovic, & MacPherson, 2011). In the trauma literature, impulsivity has also frequently been studied together with anger (e.g., Chemtob, Hamada, Roitblat, & Muraoka, 1994). However, these two concepts seem to be independent of each other. Given that impulsivity is seen as the opposite of self-control, studying self-control in the context of traumatization would be another promising approach.

With the exception of self-esteem, we found only a few studies that treat self-efficacy, locus of control, and impulsivity as dependent variables after a traumatic event. According to the model of shattered assumptions (Janoff-Bulman, 1992), after a traumatic event, individuals may perceive the world as being hostile and meaningless and the self as being damaged and worthless. More recent models of PTSD emphasize the importance of such dysfunctional thoughts and attitudes in trauma victims, as these may hinder their recovery from the disease (Ehlers & Clark, 2000; Foa & Rothbaum, 1999).

Given that a considerable amount of research has provided evidence for the positive impact of motivation on mental health (Forstmeier & Maercker, 2008; Heckhausen, Wrosch, & Schulz, 2010), it is of particular importance to know whether and how traumatic stress impacts motivation. Future research is required to shed light on such mechanisms.

In accord with the findings reported by Luszczynska et al. (2009), we conclude that the role of motivational variables as mediators or moderators remains unclear. First, very few studies have examined this relationship. Second, the predictor and criteria variables differed across the studies. The studies reviewed here examined the mediation hypothesis, that is, whether individuals who have survived traumatic events also tend to report lower motivation, which, in turn, has an impact on the development of psychopathology. Due to contradictory results from existing studies, more research is needed to clarify these processes.

Compared with the other three constructs, self-esteem has been investigated more frequently in younger populations. Of particular interest is the finding that associations between traumatic stress and self-esteem in children and adolescents are considerably weaker than those same relationships in adults. This difference may be a result of the various types of trauma (e.g., maltreatment in children vs. war combat in adults) or may be due to comparably higher resilience in children (Masten, 2007). Another possibility has its origins in developmental psychology and argues that children may process traumatic contents differently than adults, which leads to differing characteristics of PTSD (Maercker, Michael, Fehm, Becker, & Margraf, 2004; Pynoos, Steinberg, & Piacentini, 1999). Thus, the impact of a traumatic event might have more serious damage in adulthood than in childhood due to the more advanced development of motivations, cognitions, emotions, and behaviors in adults. The difference between adults and children may further be due to biased self-reports in maltreated young and very young participants (Eisen, Goodman, Qin, Davis, & Crayton, 2007).

Very few studies have tested gender effects, and those that have offer ambiguous findings. Studies with traumatized children report comparable levels of self-esteem in girls and boys. There is evidence that self-esteem in children is generally high for both genders. Given that self-esteem develops parallel to cognitive maturation, it declines during adolescence and gender divergences become more salient (Robins & Trzesniewski, 2005). Research suggests that the small significant gender difference in self-esteem in adults may be due to different sources that are important for self-esteem in women and men (Kling, Shibley Hyde, Showers, & Buswell, 1999). Therefore, it is possible that in the trauma context, as for self-efficacy, the trauma type influences a change in self-esteem. This may also be true for the full range of motivation-related constructs. However, similar to Luszczynska et al. (2009), statements about the magnitude of gender effects in self-efficacy, locus of control, self-esteem, and impulsivity cannot be made from our literature review.

### Limitations of existing research

In summary, studies examining the role of motivational concepts in trauma research use different samples, trauma types, study designs, and measuring instruments. Thus, a direct comparison of the results from these heterogeneous data is complicated. One reason for the inconsistency in results could be the angle from which different studies view the relationship between trauma exposure and motivational variables. For example, in the context of stressful life events, the association between situation-specific measures and an outcome tend to be stronger compared with the relationship between general measures and an outcome (Frazier et al., 2011). Given this difference, a comparison of the reviewed studies’ findings is further complicated by the factors, such as self-efficacy, that are used as general and situation-specific variables.

In the majority of the studies, young adults and children were the most frequently examined subjects. With regard to the other end of the lifespan, only two studies considered self-efficacy and locus of control in an elderly sample. Not only has research neglected the occurrence of posttraumatic stress in older populations, but it has also ignored the development of a delayed-onset of this disorder (Averill & Beck, 2000). Given evidence that PTSD may increase the risk for dementia (Yaffe et al., 2010) and that motivational variables predict memory performance in older adults (Forstmeier & Maercker, 2008; Valentijn et al., 2006), it is of great importance to discover the mechanisms that operate between traumatic experiences and motivation.

With the exception of studies on impulsivity, which only focused on individual traumas, studies with the three other constructs examined both collective and individual traumas. Regardless of the type of trauma, no differences concerning the relationship between traumatic stress and motivational factors were found. Even the same type of trauma (e.g., man-made or accidental) does not seem to generate an obvious pattern concerning the association between trauma exposure and motivation-related outcomes. This question remains to be answered.

Finally, expectancy beliefs that have an impact on goal pursuit are underrepresented as outcome variables in the trauma research. In accord with empirical evidence that personally meaningful goals have a beneficial effect on well-being (Brunstein et al., 1999), it seems essential to know the influence of traumatic stress on motivation.

### Future directions

Recent research provides evidence that self-efficacy is considered to be one of the central factors in attaining posttraumatic recovery (Benight & Bandura, 2004; Benight, Ruzek, & Waldrep, 2008). Accordingly, promoting self-efficacy would contribute to encouraging individuals with trauma exposure to dare to engage in less avoidance and explore potentially corrective experiences. Furthermore, attributional elements of the motivational constructs should be included in treatments with trauma victims. Several studies suggest that the helplessness concept has proven itself a useful model in the context of PTSD. That is, more PTSD symptoms seem to be related to a more pronounced disadvantageous attributional style (Elwood, Hahn, Olatunji, & Williams, 2009; McKeever, McWhirter, & Huff, 2006; McCormick, Taber, & Kruedelbach, 1989; Palker-Corell & Marcus, 2004). Moreover, there are some indices that individuals with trauma exposure face difficulties when they engage in goal-directed behavior in stressful situations (Weiss et al., 2012). Therefore, applying more favorable attributional style patterns may lead to improved motivation (e.g., by stimulating approach motivation) and, in turn, to better recovery from PTSD. The concept of approach and avoidance motivation, which has primarily been studied in motivational psychology, should be incorporated into future trauma research (Elliot, 2008; Grawe, 2007). Approach-motivated individuals show behavioral tendencies that are directed by a desirable event, which serves to need satisfaction. In contrast, individuals who pursue avoidance goals are primarily motivated to avoid need frustration. Avoidance motivation is very pronounced in trauma victims. Therefore, it is necessary to include motivational components in the treatment of trauma sequelae.

Finally, the cognitive processing therapy for PTSD (Resick & Schnicke, 1992), which has been proven effective in several studies (Monson et al., 2006; Resick et al., 2008), includes inter alia the challenging of overgeneralized beliefs in different areas, such as safety, trust, power, control, esteem, and intimacy. The motivational constructs discussed here (e.g., self-efficacy, locus of control, and impulsivity) can be linked to maladaptive thoughts about control and esteem (e.g., self-esteem). Therefore, changing such dysfunctional convictions can help individuals to successfully recover from a trauma.

## Conclusion

To conclude, we summarized and discussed the literature on motivation (self-efficacy, locus of control, self-esteem, and impulsivity) in individuals with a history of trauma. The existing research on the relation between traumatic stress and motivation faces some challenges. First, it is clear that more studies with longitudinal designs are needed. Presently, the majority of longitudinal studies in this area have tested the role of self-efficacy in the trauma context. It is desirable to have longitudinal studies that examine the effects of additional motivational constructs as well. Second, motivation-related constructs should be considered as outcome variables more frequently. Studies should investigate whether trauma exposure leads to a change in these variables. Third, the role of motivation as a mediator or moderator should be included in future analyses. Fourth, more studies of old-aged individuals are required, so that a perspective regarding the entire life span can be provided. Fifth, the stabilizing and strengthening of personal expectancies (i.e., self-efficacy, locus of control, and self-esteem) and self-control in trauma victims should become the main goal of treatment programs, as these are essential for goal setting and goal implementation, which are of great importance for individual well-being and life adjustment.
